# High Prevalence of Posttraumatic Stress Disorder Symptoms Found in Well-Treated People with HIV after the Introduction of Patient-Reported Outcome Measures

**DOI:** 10.1007/s10461-025-04617-x

**Published:** 2025-01-27

**Authors:** Kevin Moody, Colette Smit, Pythia T. Nieuwkerk, Maarten Bedert, Elise Nelis, Jeannine Nellen, Kim Sigaloff, Annouschka Weijsenfeld, Laura Laan, Claire Bruins, Suzanne E. Geerlings, Marc van der Valk

**Affiliations:** 1 Division of Infectious Diseases, Amsterdam University Medical Center, University of Amsterdam, G7, Meibergdreef 9, Amsterdam, 1105 AZ The Netherlands; 2 Amsterdam Institute for Immunology and Infectious Diseases (AII), Amsterdam University Medical Center, Amsterdam, The Netherlands; 3 Amsterdam Public Health Research Institute, Amsterdam University Medical Center, Amsterdam, The Netherlands; 4 Stichting HIV Monitoring, Amsterdam, The Netherlands; 5 Department of Medical Psychology, Amsterdam University Medical Center, University of Amsterdam, Amsterdam, The Netherlands; 6 Knowledge and Care Center for Gender Dysphoria, Amsterdam University Medical Center, Vrije Universiteit, and Kaleidos LGBT+ Mental Health Center, Amsterdam, The Netherlands; 7 Division of Infectious Diseases, Amsterdam University Medical Center, Vrije Universiteit, Amsterdam, The Netherlands

**Keywords:** Posttraumatic stress disorder, Mental Health, Quality of life, Patient reported outcome measures, Europe < region

## Abstract

People with HIV (PWH) are at greater risk of experiencing mental health problems, such as depression and post-traumatic stress disorder (PTSD). The purpose of our study was to determine the prevalence of posttraumatic stress disorder PTSD in PWH. PWH in care Amsterdam University Medical center (Amsterdam UMC) with access to the electronic patient portal were offered patient-reported outcome measures (PROMs) between May 2022 and May 2023, including the PC-PTSD-5 screen for PTSD as part of routine clinical care. Risk factors for a clinically relevant PC-PTSD-5 score were determined using univariate and multiple logistic regression analyses. Of 2476 PWH included, 1384 (55.9%) had access to the electronic patient portal of whom 474 (34.2%) completed the PC-PTSD-5. PWH without access to the patient portal were more often female, born in low- and middle-income countries, acquired HIV more often via heterosexual contact, and had worse HIV-related outcomes compared to those with access. Of 474 PWH who completed the PC-PTSD-5 screening question, 62 (13.1%) reached the threshold for clinically relevant PTSD. Age less than 50 years (OR 2.29, 95% CI: 1.21–4.35), and having originated from low- or middle-income countries (OR 2.02, 95% CI: 1.09–3.76) were associated with PTSD. Our findings show that 13% of PWH with a well-controlled HIV infection with access to the electronic patient portal at Amsterdam UMC experienced clinically relevant PTSD complaints.

**Supplementary Information:**

The online version contains supplementary material available at 10.1007/s10461-025-04617-x.

## Introduction

HIV infection has become a chronic illness and currently the life expectancy of people with HIV (PWH) approaches that of the general population [1]. However, mental health is a major outstanding problem. PWH are at greater risk of experiencing mental health problems, such as depression and posttraumatic stress disorder (PTSD), which may affect clinical outcomes and quality of life, and can be predictors for non-adherence to treatment [2, 3]. Receiving an HIV diagnosis can in itself be a source of trauma [4]. PTSD and HIV can occur sequentially or simultaneously, but these conditions can also interact with each other. Both are associated with similar risk factors, like drug use, risky sexual behaviours, low socioeconomic status, and experienced trauma [5]. Furthermore, HIV and mental health problems can also mutually affect each other when they co-exist, which further complicates diagnosis and treatment [6, 7]. 

The *Diagnostic and Statistical Manual of Mental Disorders*,* volume 5* outlines criteria for diagnosing PTSD based on exposure to a traumatic event, presence of symptoms related to and occurring after the event, avoidance of stimuli associated with the event, and negative changes in thinking and mood [8]. In the general population, the one-year prevalence of PTSD among individuals exposed to trauma is estimated to be 5.6% [9]. Two systematic reviews have confirmed that the global pooled prevalence of PTSD is higher in PWH (28% and 32.7%) and that various instruments have been shown to effectively detect PTSD in clinical and research settings [10, 11]. These studies were conducted mostly in countries without universal health care access and/or not in a context of well-treated PWH; it is currently not known to what extent PTSD is a relevant problem among well-treated PWH.

The PC-PTSD-5 is a patient-reported outcome measure (PROM) that has been shown to detect PTSD symptoms in PWH [12]. PROMs, including the PC-PTSD-5, were introduced at Amsterdam UMC in 2022 as part of routine clinical care to screen for PTSD symptoms.

The purpose of our study was to determine the prevalence of PTSD in PWH at Amsterdam UMC and explore associations with clinical and demographic characteristics.

## Methods

### Setting

This study involved PWH engaged in care at Amsterdam UMC. Since May 2022, patient-reported outcome measures– including for PTSD– were introduced at the HIV outpatient clinics at Amsterdam UMC [13]. We limited the analyses to individuals who had consented to data collection as part of the ongoing AIDS Therapy Evaluation in the Netherlands (ATHENA) cohort for which 98% of all PWH engaged in care in the Netherlands have provided consent [14]. Pseudonymized data transfer and analysis mechanisms for these individuals are managed by Stichting HIV Monitoring on behalf of the ATHENA cohort through agreements with all treatment centres in the Netherlands [15]. 

### Study Procedures

PROMs were sent one week prior to their consultation as part of routine clinical care to all people engaged in care aged 18 years and above who could interact with health care providers (HCP) in either English or Dutch and who were registered with the electronic patient portal (My Chart) from May 9, 2022 to May 15, 2023. During the annual consultation, HCP and PWH discussed PROMs scores and determined follow-up activities if needed.

### PROMs Selected for Routine Clinical Care

PROMs for routine clinical care include the PC-PTSD-5 for posttraumatic stress disorder, a short instrument comprising a screening question, followed by five items for positive screens [16] (see Supplement, Appendix 1). Also, as reported elsewhere, the following PROMs were offered: PROMIS Computer Adaptive Tests (CAT) for anxiety (version 1.0), depressive symptoms (version 1.0), fatigue (version 1.0), sleep disturbances (version 1.1), social isolation (version 1.0), physical functioning (version 1.2) [17]; Berger HIV Stigma Scale subdomains for negative self-image and disclosure issues [18]; Medication Adherence Report Scale [19]; adapted Alcohol Use Disorder Identification Test [20]; a substance questionnaire based on the Drug Use Disorder Identification Test [21]; and bespoke questionnaires about sexual health, and issues related to finances, housing, and migration status [13]. 

### Analysis and Statistical Considerations

The primary endpoint was clinically relevant PTSD symptoms defined as a PC-PTSD-5 score of 3 or higher [16]. PWH who indicated that they had never experienced a traumatic event in the screening question were added to the group who completed the entire questionnaire and scored lower than 3.

Descriptive data included PC-PTSD-5 scores, demographics– age, sex, region of origin– and HIV-specific characteristics– years since diagnosis, years on treatment, route of transmission, most recent HIV-1 RNA and CD4 count, nadir CD4 and anchor drug of antiretroviral treatment (ART) at time of completion of the questionnaires.

First, we compared the demographic and clinical characteristics of PWH at Amsterdam UMC during the study period who did and did not have access to the electronic patient portal, using Man-Witney or Pearson’s chi squared tests as appropriate. The purpose of this comparison was to examine whether our sample was representative of the total PWH population. Second, we compared demographic and clinical characteristics of PWH with access to the patient portal who did and did not complete the PC-PTSD-5.

For those who completed the PC-PTSD-5, we identified socio-demographic/ HIV-specific characteristics significantly associated with clinically relevant PTSD symptoms, using a series of binary logistical models. Socio-demographic/ HIV-specific characteristics were added one by one as fixed factors to the model. The presence of clinically relevant PC-PTSD-5 scores of 3 or higher (yes/no) was the dependent variable. Socio-demographic/ HIV-specific characteristics with a Wald χ^2^ test p-value < 0.20 were included in further multivariable modelling. Subsequently, socio-demographic/ HIV-specific characteristics with p-values > 0.05 were removed from the multivariate model using backward elimination.

ART anchor drugs might influence PTSD symptoms and could be potential confounders. We therefore added each individual integrase, non-nucleoside or protease inhibitor used at the time of the assessment one by one to our final multivariable model. An ART anchor drug was considered a confounder if it changed the effect estimate of the most significant variable by 10%. We reported both the unconfounded and confounded results for compounds that reached this threshold.

We reported the number and percentage of PWH with moderate to severe symptoms of anxiety, depression, fatigue, sleep disturbances, social isolation, and physical functioning on PROMIS-CAT questionnaires, stratified by clinically relevant PTSD. T-scores equal to or greater than 60 (1 standard deviation above the mean) functioned as cut off scores for these domains, except for physical functioning, where this was less than 40 (1 standard deviation below the mean) [22]. We reported raw Berger HIV Stigma Scale scores for the subdomains of disclosure concerns and negative self-image. Higher scores for both sub-scales indicate worse stigma in those domains [23]. We indicated the number and percentage of PWH who reported problems with housing, finances and/or migration status based on a bespoke 1-item screening question, “Do you experience any problems regarding housing, income and/or legal status? (Yes/No)”.

Two-sided p-values < 0.05 are considered to indicate statistical significance. Data analyses were conducted using SPSS version 26 and/or Stata version 16.

### Ethical Approval

Participants consented to participate in the analysis of data collected as part of routine clinical care to the AIDS Therapy Evaluation in the Netherlands study (ATHENA) cohort through mechanisms described in Boender et al. [15] Additional ethical approval for the analysis of these data was not required under the ATHENA cohort protocol [13]. 

## Results

In total, 2476 PWH were included in our study, of whom 1384 (55.9%) had access to the electronic patient portal during the study period. PWH without access to the patient portal were more often female, born in low- and middle-income countries, acquired HIV more often via heterosexual contact or IV drug use, had lower current and nadir CD4-counts, and were less often virally suppressed compared to PWH with access to the portal. (Table 1)

**Table 1:** Comparison of demographic and clinical characteristics of people with HIV in care during the study period, patients with and without access to the patient portal and those who did and did not complete the PC-PTSD-5 PROMs

	Total in Care^†^ *n*(%)	Access to Patient Portal *n*(%)/median (IQR)	No access to Patient Portal *n*(%)/median (IQR)	*P*-value	Completed PC-PTSD-5 *n*(%)/median (IQR)	Did not complete PC-PTSD-5 *n*(%)/median (IQR)	*P*-value
**Total**	2764	1384 (55.9)	1092 (44.1)		474 (34.2)	910 (65.8)	
*Demographic*							
Age (years)	53 (43–61)	53 (42.25-61)	53 (44.25-61)	0.060^‡^	55 (45–64)	52 (41.75-59)	< 0.001^‡^
Sex	1851 (74.8)	1142 (82.5)	709 (64.9)	< 0.001^§^	404 (85.2)	738(81.1)	0.055^§^
	625 (25.2)	242 (17.5)	383 (35.1)		70 (14.8)	172 (18.9)	
Country/ Region of birth	1093 (44.1)	808 (58.4)	285 (26.1)	< 0.001^§^	336 (70.9)	472 (51.9)	< 0.001^§^
	155 (6.3)	104 (7.5)	51 (4.7)		41 (8.6)	63 (6.9)	
	1182 (47.7)	446 (32.2)	736 (67.4)		97 (20.5)	349 (38.4)	
	46 (1.9)	26 (1.9)	20 (1.8)		0	26 (2.9)	
*Clinical*							
Years since HIV diagnosis	16 (10–22)	16 (10–22)	16 (11–22)	0.368^‡^	16 (10–23)	16 (9–22)	0.073^‡^
Duration on HIV treatment (years)	14 (9–20)	14 (8–20)	14 (9–21)	0.094^‡^	14 (9–21)	14 (8–20)	0.122^‡^
Route of HIV transmission	1280 (51.7)	906 (65.5)	374 (34.2)	< 0.001^§^	351 (74.1)	555 (61.0)	< 0.001^§^
	1095 (44.2)	437 (31.6)	658 (60.3)		114 (24.1)	323 (35.5)	
	101 (4.1)	41 (3.0)	60 (5.5)		9 (1.9)	32 (3.5)	
HIV-1 RNA (last as of 31/12/2022)	2345 (94.7)	1329 (96.0)	1016 (93.0)	< 0.001^§^	463 (97.7)	866 (95.2)	0.075^§^
	55 (2.2)	16 (1.2)	39 (3.6)		3 (1.4)	13 (1.4)	
	76 (3.1)	39 (2.8)	37 (3.4)		8 (1.7)	31 (3.4)	
CD4-count (cells/mm^3^) (last as of 31/12/2022)	690 (510–910)	705 (550–910)	670 (480–910)	0.016^‡^	690 (537.5–900)	710 (550–910)	0.591^‡^
Nadir CD4 (cells/mm^3^)	220 (100–360)	240 (120–380)	200 (80–340)	< 0.001^‡^	240 (130–377)	240 (118.75–380)	0.512^‡^

^†^Total in care refers to the total number of individuals in care who consented to the ongoing AIDS Therapy Evaluation in the Netherlands (ATHENA) cohort, ^‡^Mann-Whitney, ^§^Pearson Chi-Square

Out of the 1384 individuals with access to the electronic patient portal, 474 (34.2%) completed the PC-PTSD-5. PWH who completed the PTSD questionnaire were slightly older, more likely to be of Dutch origin, and were more likely men who have sex with men (MSM) compared to those with access to the portal who did not complete the questionnaire.

Of the 474 individuals who completed the PC-PTSD-5, 62 (13.1%) reported clinically relevant PTSD symptoms. (Table 2) In multivariable analyses, younger age (OR: 2.29, CI 95%: 1.21–4.35), and having been born in a low- or middle-income country as compared to having been born in the Netherlands (OR: 2.02, CI 95%: 1.09–11.43), were significantly associated with clinically relevant PTSD symptoms. Viral suppression was not statistically significantly different between the two groups. (Table 3) None of the ART anchor drugs changed the estimates more than 10%.

**Table 2:** Characteristics of people with HIV by clinically relevant PTSD score

		Total *n*(%)	Without PTSD (score < 3) *n*(%)/median (IQR)	With PTSD (score³3) *n*(%)/median (IQR)	*P*-value
**Total**		474	412 (86.9)	62 (13.1)	
Age (years)		55 (44.5)	56 (46–64)	46 (40-54.25)	< 0.00^†^
Sex		404 (85.2)	357 (86.7)	47 (75.8)	0.025^‡^
Country/ Region of birth	Netherlands	335 (70.7)	300 (72.8)	35 (56.5)	0.003^iii^
	High Income Countries^§^	41 (8.6)	37 (9.0)	4 (6.5)	
	Low- & Middle-Income Countries^§^	98 (20.7)	75 (18.2)	23 (37.1)	
Years since HIV diagnosis		16 (10–23)	17 (10–23)	14 (8.75–21.25)	0.043^†^
Duration on HIV treatment (years)		14 (9–21)	15 (10–21)	12 (8–21)	0.019^†^
Route of HIV acquisition	MSM^¶^	355 (74.9)	312 (75.9)	43 (69.4)	0.266 ^‡^
	Other	118 (24.9)	99 (24.1)	19 (30.6)	
	Missing	1 (0.0)	1 (0.2)	0	
Current HIV-1 RNA	< 200 c/ml	468 (98.7)	408 (99.0)	60 (96.8)	0.139^‡^
Current CD4-count (cells/mm^3^)		710 (535–910)	700 (522.5–910)	780 (605–915)	0.080^†^
Nadir CD4 (cells/mm^3^)		240 (130–378)	230 (120–370)	277 (200–410)	0.042^†^
*ART Anchor Drugs* ^††^					
Integrase Inhibitors		262 (63.6)	305 (63.8)	43 (69.4)	
BIC			82 (31.3)	19 (44.2)	
CAB			13 (5.0)	4 (9.3)	
DTG			86 (32.8)	12 (27.9)	
EVG/c			62 (23.7)	7 (16.3)	
RAL			19 (7.3)	1 (2.3)	
Protease Inhibitors		54 (11.3)	46 (11.2)	8 (12.9)	
ATV			6 (13.0)	2 (25.0)	
DRV			40 (87.0)	6 (75.0)	
Non-nucleoside Reverse Transcriptase Inhibitors		119 (24.9)	109 (26.5)	10 (16.1)	
DOR			20 (18.3)	3 (30.0)	
EFV			31 (28.4)	2 (20.0)	
NVP			41 (37.6)	2 (20.0)	
RPV			17 (15.6)	3 (30.0)	
No ART			3 (0.7)	1 (1.6)	

^†^Mann-Whitney

^‡^Pearson Chi-Square

^§^High income countries and Low- and middle-income countries as defined by the World Bank (https://datatopics.worldbank.org/world-development-indicators/the-world-by-income-and-region.html)

^¶^MSM: men who have sex with men

^††^BIC = bictegravir, CAB = cabotegravir, DTG = dolutegravir, EVG/c = elvitegravir and cobicistat, RAL = raltegravir, ATV = atazanavir, DRV/c = darunavir and cobicistat, DOR = doravirine, EFV = efavirenz, NVP = nevirapine, RPV = rilpivirine

**Table 3:** PTSD Univariate and multivariate logistic regression

		Univariate			Multivariable	
	Odds Ratio	CI 95%	p-value^†^	Odds ratio	CI 95%	p-value^†^
Age < 50 years versus > 50 years	38.1	1.84–5.49	**< 0.001**	2.29	1.21–4.35	**0.011**
Male versus female	2.07	1.09–3.96	**0.027**	1.67	0.85–3.31	0.139
Region Netherlands (reference)	1		**0.004**	1		0.071
Not-Netherlands, High-income countries	0.93	0.31–2.75	0.891	0.916	0.30–2.77	0.876
Not-Netherlands, Low- and Middle-income countries	2.63	1.47–4.71	**0.001**	2.02	1.09–3.76	**0.026**
Years since diagnosis < 14 versus = > 14 (median)	0.65	0.39–1.10	**0.110**	1.05	0.57–1.95	0.879
Years on treatment < 14 versus = > 14 (median)	0.59	0.34-1.00	**0.052**			
Transmission Route MSM versus other	0.72	0.40–1.29	0.268			
VL < 200 versus > 200	3.40	0.61–18.97	0.163			
CD4 < 200 versus > 200	0.600	0.07–5.46	0.650			
Nadir < 200 versus > 200	2.23	1.19–4.18	**0.012**	1.76	0.89–3.49	0.107

^†^Wald χ^2^ test

Notes

Co-variates with univariate analysis p-value = < 0.20 taken into the multivariable analysis

Time since diagnosis and years on treatment highly correlated (Pearson 0.798). Time since diagnosis taken into the model due to its greater effect

When comparing PWH with and without PTSD symptoms on other PROMS, individuals with PTSD reported significantly higher percentages of moderate to severe scores of the following PROMIS CAT domains (Table 4): anxiety (50.8%, compared to 18.0%); depressive symptoms (47.5%, compared to 10.2%), fatigue (45.2%, compared to 12.6%); sleep disturbances (30.6%, compared to 12.4%); and social isolation (21.0%, compared to 5.1%). All the aforementioned PROMs were significantly correlated with PTSD scores, and among themselves. Berger HIV Stigma Scale scores for the subdomain of negative self-image were significantly higher for the PTSD group (6.30, SD 2.49), compared to the non-PTSD group (5.72, SD 2.07). 32% of PWH with PTSD symptoms reported problems with housing, finances, and/or migration status, compared to 8.9% in the non-PTSD group.

**Table 4:** Patient-reported outcomes in individuals with and without clinically relevant PTSD scores^†^

		Total *n*(%)	Without PTSD *n*(%)	With PTSD *n*(%)	*P*-value
PROMIS CAT scores					
Anxiety (*n* = 471)	Moderate to Severe	105 (22.3)	74 (18.0)	31 (50.8)	< 0.001^‡^
	None to Mild	366 (77.7)	336 (82.0)	30 (49.2)	
Depressive symptoms (*n* = 471)	Moderate to Severe	71 (15.0)	42 (10.2)	29 (47.5)	< 0.001^‡^
	None to Mild	400 (84.9)	368 (89.8)	32 (52.5)	
Fatigue (*n* = 473)	Moderate to Severe	80 (16.9)	52 (12.6)	28 (45.9)	< 0.001^‡^
	None to Mild	393 (83.1)	360 (87.4)	33 (54.1)	
Sleep disturbances (*n* = 473)	Moderate to Severe	70 (14.8)	51 (12.4)	19 (31.1)	< 0.001^‡^
	None to Mild	403 (85.2)	361 (87.6)	42 (68.9)	
Social isolation (*n* = 468)	Moderate to Severe	34 (7.3)	21 (5.1)	13 (21.7)	< 0.001^‡^
	None to Mild	434 (92.7)	387 (94.9)	47 (78.3)	
Physical Functioning (*n* = 473)	Moderate to Severe	76 (16.1)	58 (14.1)	18 (29.5)	0.002^‡^
	None to Mild	397 (83.9)	58 (14.1)	18 (29.5)	
Other					
Berger HIV Stigma Scale, disclosure concerns Mean (SD)			8.60 (2.50)	8.90 (2.93)	0.385^§^
Berger HIV Stigma Scale, negative self-image Mean (SD)			5.72 (2.07)	6.30 (2.49)	0.005^§^
Problems with financial, housing and/or migration status N (%)		53 (11.3)	36 (8.9)	17 (27.9)	< 0.001^‡^

^†^ Clinically relevant PTSD scores defined as scores ≥ 3 on the PT-PTSD-5 questionnaire

^‡^ Pearson Chi-Square

^§^ Independent samples T test

## Discussion

We found that 13% of PWH with well-controlled HIV at Amsterdam UMC who had access to the electronic patient portal had an indication of active, clinically relevant PTSD symptoms, which were associated with being younger and having been born in a low- or middle-income country. As far as we know, our study is the first to examine PTSD prevalence in well-treated PWH in out-patient HIV clinics as part of routine clinical care using a short-form PTSD screening instrument.

Our finding that clinically relevant PTSD was more prevalent in young people is consistent with other studies that show that mental health problems decrease as people age [10, 11]. We found significant correlations between PTSD and all patient-reported scores for anxiety, depression, fatigue, sleep disturbances, and social isolation and PTSD, as well as significant correlations among these PROMs scores.

Poor socioeconomic status can heighten vulnerability through migration status, language barriers, unstable housing and financial instability [24, 25], and can contribute to a higher prevalence of mental health issues, including PTSD [26]. People born in low- and middle-income countries in our study reported a higher prevalence of clinically relevant PTSD, which is consistent with findings from a systematic review of PTSD in PWH [10]. Furthermore, almost one-third of people with PTSD in our study reported problems with housing, finances and/or migration status, with half of those originating from low- and middle-income countries.

Healthcare providers (HCP) play a pivotal role in the mental health of PWH through detection, shared decision-making surrounding follow-up activities, and referrals where necessary. However, some HCP might feel they lack the training necessary to detect and triage mental health issues in PWH [2]. This is problematic, given the high prevalence of mental health issues in PWH and the primacy of the PWH-HCP relationship.

HIV HCP are often the primary contact for PWH in the health system. Trust is cultivated between PWH and HCP through the years, and HCP’s flexibility and perception of PWH vulnerability further strengthen that relationship [27–30]. This trust is not necessarily extended by PWH to HCP outside of HIV care. In the Netherlands, PWH report increased enacted stigma towards PWH from non-HIV HCP [31]. This is consistent with studies from other countries where people with a migration background have reported similar experiences [32]. Perceived stigma from the community can also play a role. In exploratory research looking at the patient journey in our clinic, PWH from Ghana and Nigeria reported feeling safe only when sitting across from their HCP and often do not disclose their HIV further [33]. 

Consequently, HIV clinics should consider introducing routine screening protocols for PTSD and other mental health conditions, coupled with referrals to health services and/or peer support when necessary. These measures aim to streamline the process of identifying and prioritising mental health issues among PWH, thereby improving care within HIV clinical settings, reducing the risk of non-adherence and, ultimately, improving the quality of life of PWH.

### Strengths and Limitations

Strengths of our study include its unique context: a large cohort of well-treated PWH in a setting of universal access to care screened for PTSD. Furthermore, the PTSD screening was part of a comprehensive mental health screening through the use of PROMs as part of routine clinical care. Furthermore, our population was diverse with almost half having been born in low- and middle-income countries, which exposed important disparities within our population.

Our study presents some important limitations. Only PWH with access to the electronic patient portal were offered the PC-PTSD-5 in either English or Dutch as part of routine clinical care. That means that people who could not speak these languages, those who had low literacy, and those with limited digital literacy were not included in the analyses. This includes PWH with structural vulnerabilities and/or with a migration background and, as such, it is likely that our study underestimated the prevalence of PTSD in the whole population of PWH at Amsterdam UMC. This is reflected in our comparison of groups with and without access to the electronic patient portal, which shows that our analysis lacks sufficient representation of females, those who acquired HIV through heterosexual sex or intravenous drug use, and people born in low- and middle-income countries, which is a group with a higher odds for having clinically relevant PTSD. The worse clinical outcomes of this group– less often supressed viral load and lower current and nadir CD4-counts– illustrate the potential need for additional attention to PTSD screening in this population.

The PC-PTSD-5 is a short screening tool that is easily implemented in the clinic setting; however, it does not provide a clinical diagnosis. Therefore, clinicians must further assess PWH who score ≧ 3 on the PC-PTSD-5 to determine if they require more information and/or referral to services to treat PTSD. Therefore, a mental health roadmap as a decision-support tool to support HIV HCP to facilitate care and referral for PWH with clinically relevant PTSD symptoms should be implemented to ensure adequate care.

## Conclusions

We found that 13% of well-treated PWH with access to the patient portal at Amsterdam UMC have clinically relevant PTSD symptoms, and that being younger or having been born in a low- or middle-income country, are significantly associated with PTSD symptoms. We suspect that this is an underestimation of prevalence because only PWH with access to the electronic patient dossier were offered questionnaires. We recommend that PWH should be routinely screened for PTSD as part of routine clinical care. Through a process of shared decision making, HCP and PWH should discuss whether follow-up actions are necessary and, if so, whether informal services, such as peer support or professional mental health services are required to address their PTSD and any other comorbid mental health issues. Sufficient training and support must be provided to HCP to be able to undertake these tasks.

## Electronic Supplementary Material

Below is the link to the electronic supplementary material.

Supplementary Material 1

## Data Availability

Authors agree to make data and materials supporting the results or analyses presented in their paper available upon reasonable request. It is up to the author to determine whether a request is reasonable.
