# Supplementary material for: High Prevalence of Posttraumatic Stress Disorder Symptoms Found in Well-Treated People with HIV after the Introduction of Patient-Reported Outcome Measures
Source: AIDS Behav. 2025 Jan 27;29(5):1470–8. doi: 10.1007/s10461-025-04617-x (PMC12031931; doi:10.1007/s10461-025-04617-x)
Supplement: Supplementary file 1 — Supplementary Material 1 [file 10461_2025_4617_MOESM1_ESM.docx]

**Supplementary Information**

Self-reported screening tool introduced in routine clinical care detects high levels of posttraumatic stress disorder symptoms in well-treated people with HIV. AIDS and Behaviour. Kevin Moody, Colette Smit, Pythia T Nieuwkerk, Maarten Bedert, Elise Nelis, Jeannine Nellen, Kim Sigaloff, Annouschka Weijsenfeld, Laura Laan, Claire Bruins^,^ Suzanne E Geerlings, Marc van der Valk. Correspondance: k.moody@amsterdamumc.nl

Online resource 1: PTSD-PC-5 Instrument^[[1]](#footnote-1)^

| Sometimes things happen to people that are unusually or especially frightening*,*horrible*,*or traumatic. For example*: •*a serious accident or fire *•*a physical or sexual assault or abuse *•*an earthquake or flood *•*a war *•*seeing someone be killed or seriously injured *•*having a loved one die through homicide or suicide  Have you ever experienced this kind of event*?* | YES/NO |
| --- | --- |
| If ‘No,’ screen total = 0; if ‘Yes,’ continue with screening. In the past month, have you… | |
| 1. Had nightmares about the event(s) or thought about the event(s) when you did not want to? | YES/NO |
| 2. Tried hard not to think about the event(s) or went out of your way to avoid situations that reminded you of the event(s)? | YES/NO |
| 3. Been constantly on guard, watchful, or easily startled? | YES/NO |
| 4. Felt numb or detached from people, activities, or your surroundings? | YES/NO |
| *5.*Felt guilty or unable to stop blaming yourself or others for the events*(*s*)*or any problems the event*(*s*)*may have caused*?* | *YES/NO* |

1. Prins A, Bovin MJ, Smolenski DJ, Marx BP, Kimerling R, Jenkins-Guarnieri MA, et al. The Primary Care PTSD Screen for DSM-5 (PC-PTSD-5): Development and Evaluation Within a Veteran Primary Care Sample. J Gen Intern Med. 2016;31(10):1206-11. [↑](#footnote-ref-1)
